# Gastrointestinal Histoplasmosis: A Descriptive Review, 2001–2021

**DOI:** 10.3390/life13030689

**Published:** 2023-03-03

**Authors:** Bassey E. Ekeng, Asa E. Itam-Eyo, Iriagbonse I. Osaigbovo, Adilia Warris, Rita O. Oladele, Felix Bongomin, David W. Denning

**Affiliations:** 1 Medical Mycology Society of Nigeria, Lagos 101017, Nigeria; 2 Department of Medical Microbiology and Parasitology, University of Calabar Teaching Hospital, Calabar 540271, Nigeria; 3 Department of Internal Medicine, University of Calabar Teaching Hospital, Calabar 540271, Nigeria; 4 Department of Medical Microbiology, School of Medicine, College of Medical Sciences, University of Benin, Benin City 300213, Nigeria; 5 Medical Research Council Centre for Medical Mycology, University of Exeter, Exeter EX4 4QD, UK; 6 Department of Medical Microbiology and Parasitology, Faculty of Basic Medical Sciences, College of Medicine, University of Lagos, Lagos 101017, Nigeria; 7 Department of Medical Microbiology & Immunology, Faculty of Medicine, Gulu University, Gulu P.O. Box 166, Uganda; 8 Manchester Fungal Infection Group, Faculty of Biology, Medicine and Health, The University of Manchester, Manchester Academic Health Science Centre, Manchester M13 9WL, UK

**Keywords:** gastrointestinal, histoplasmosis, HIV/AIDS, immunosuppressed

## Abstract

Gastrointestinal histoplasmosis (GIH) is infrequently described in people without underlying HIV infection. We aimed to compare the clinical presentation of GIH in people with and without HIV infection. We conducted a literature search of published cases of GIH from 2001–2021 and found 212 cases. Of these, 142 (67.0%) were male, and 124 (58.5%) had HIV infection. Most cases were from North America (n = 88, 41.5%) and South America (n = 79, 37.3%). Of the 212 cases, 123 (58.0%) were included in both clinical and pathological analyses. The remainder were excluded as details about clinical and pathological findings were not available. Of the 123 cases, 41 had HIV infection while 82 were without HIV infection. The diagnosis was predominantly by histopathology (n = 109, 88.6%). A significant proportion of people with HIV infection had abdominal pain as the most predominant symptom of GIH compared to those without HIV infection (65.9% versus 41.9%, *p* < 0.05). The colon was the most affected site with a slightly higher proportion in those with HIV infection compared with cases without HIV infection (46.3% versus 42.7%). The commonest pathologic findings were caecal and ileal ulcers. Caecal ulcers were significantly more frequent in cases with HIV infection compared to those without HIV (32.1% versus 7.1%, *p* < 0.05). Despite being more common in people with HIV infection, GIH also affects people without HIV infection with similar clinical presentations.

## 1. Introduction

Histoplasmosis is a serious fungal disease that occurs worldwide; endemics have occurred in the Ohio and Mississippi river valley regions of the USA and Central and South America including Guatemala, Guiana Shield, Dominican Republic, Trinidad and Tobago and Brazil [1,2]. Increasing cases are also now being reported in West Africa, Central Africa, East Africa, Southern Africa and Southeast Asia [1,3]. Histoplasmosis is primarily acquired via inhalation of *Histoplasma* microconidia aerosolised following disruption of soil rich in guano. Caving, farming, hunting, excavation and travel to endemic regions are additional risk factors [1,2]. The spectrum of clinical manifestation ranges from asymptomatic infection, acute pulmonary disease, chronic pulmonary disease and disseminated histoplasmosis (DH) [1,2]. DH is commonly seen in the immunocompromised, especially in people living with HIV/AIDS and others including organ transplant recipients, malignancies, chemotherapy, immunosuppressive agents, prolonged steroid usage and excessive environmental exposure [2,4,5,6,7].

Gastrointestinal histoplasmosis (GIH) is a rarely reported clinical form of DH. The most common sites of involvement are the colon and ileum [8,9]. Affinity for those areas of the gastrointestinal tract is attributed to the abundance of lymphoid tissues [8,9]. In an autopsy series, *Histoplasma* was identified in the gastrointestinal tract in 70% of DH cases [9]. However, symptomatic disease leading to clinical diagnosis of GIH was found in only 3–12% of patients [9]. This discordance makes it highly suspicious that GIH may be underdiagnosed due to its non-specific symptoms [9]. Besides occurring as a sequela of DH, GIH can also develop as a localised disease entity [9]. Previous reviews on GIH focused mainly on people living with HIV [8,10,11]. However, there is a paucity of studies describing the clinical presentation of GIH in people without HIV infection despite significant data showing the occurrence of GIH in immunocompetent hosts [9]. In this review, we aimed at highlighting the clinical features of GIH with a focus to compare clinical presentation and complications in people with and without HIV infection.

## 2. Materials and Methods

We conducted a literature search using PubMed, Google scholar and African Journal Online to identify case reports and case series on GIH from ‘1 January 2001–31 December 2021’ (BEE). The search term was “gastrointestinal histoplasmosis” OR “gastrointestinal AND “histoplasmosis”. References in all relevant papers were also reviewed for additional publications on case reports regarding the topic that may not have been published in the searched databases. Individual cases and case series of GIH published before 2001 were excluded. Case reports or case series without patients’ country of origin or location of disease were excluded. No language restrictions were applied. Diagnosis of histoplasmosis was defined as the identification of *H. capsulatum* var *capsulatum* or *H. capsulatum* var *duboisii* via histopathological examination of tissue samples or culture or molecular methods and/or positive *Histoplasma* antigen detection or antibody detection. A second author (AEI) independently repeated the entire search and selection process to prevent selection bias. Any inconsistencies were discussed until a consensus was reached as to what studies should be included. Data extracted from each case report included: gender, age, clinical features, pathological findings, complications, HIV infection status, immunosuppression, investigation/diagnostic measures, treatment and outcomes. A comparative analysis of the clinical features and pathological findings of GIH between cases with and without HIV infection was performed using Fisher’s exact test. A *p*-value of <0.05 was considered statistically significant.

## 3. Results

**Figure 1:**
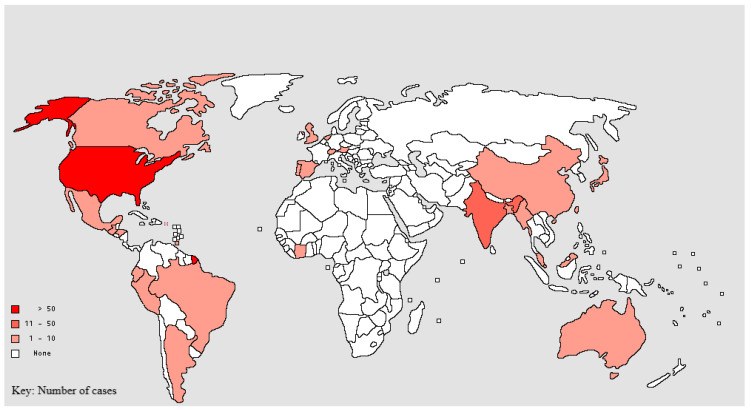
Geographical distribution of GIH cases (2001–2021).

We identified 212 cases of GIH across the world’s regions, as summarised in Table 1 and Figure 1. Of the 212 cases,142 (67.0%) were male. Only one (0.5%) case was documented in a child. In total, 124 (58.5%) cases had underlying HIV infection. GIH was predominant amongst people living with HIV from Central and South America, while more cases were seen in HIV-negative patients in North America, Asia, Europe and Australia (Table 1). Of the 88 cases without HIV infection, 15 (17.0%) were post-transplant patients on immunosuppressants, 31 (35.2%) were on immunosuppressants for various disease conditions, three (3.4%) were diagnosed with an immunodeficiency syndrome, one suffered from diabetes mellitus (0.5%) and the remaining 38 (43.1%) were immunocompetent.

Analysis of the 123 well-documented cases showed the clinical presentation of GIH was often non-specific and mimicked several other gastrointestinal conditions and pathologies, including abdominal tuberculosis (n = 3, 2.4%) [85,96,102], GIH and TB coinfection was reported in four [3.3%] cases [78,87,96,98], inflammatory bowel disease (n = 9, 7.3%) [21,33,40,52,69,75,89,105,107], intestinal obstruction (n = 15, 12.2%) [15,21,39,50,57,64,71,77,81,89,95,102,103,104,117], abdominal malignancy (n = 9, 7.3%) [14,16,31,80,83,101,102,108,112], GI bleeding (n = 29, 23.6%) [17,18,25,26,27,29,30,31,33,37,41,44,53,56,59,63,70,71,81,84,94,102,103,105,107,110,116,119], hepatitis (n = 19, 15.4%), cholecystitis (n = 7, 5.7%) [22,24,34,47,54,56,61,67,68,72,73,76,83,86,97,101,109,114,118]), acute pancreatitis (n = 3, 2.4%) [20,65,75,77] and acute abdomen (n = 4, 3.1%) [70,74,99,112]. Oesophageal involvement was rarely reported and presented mostly as ulcers or as obstruction of the oesophagus due to oesophageal polyps or mediastinal lymph nodes (n = 13, 10.6%) [13,20,40,42,56,58,77,80,100,104,116,121].

Comparing the clinical presentation between people living with HIV and people without underlying HIV infection, a significant proportion of people with HIV infection had abdominal pain as the most predominant symptom of GIH (65.9% versus 41.9%, *p* = 0.01), Table 2. The colon was the most affected site with a slightly higher proportion in those with HIV infection compared with cases without HIV infection (46.3% versus 42.7%), Table 2. The mode of diagnosis was predominantly by histopathology (n = 109, 88.6%) of biopsied specimens/tissues, as shown in Table 2. Some of the case reports highlighted endoscopy findings and are summarised in Table 3. The commonest pathological findings were caecal and ileal ulcers. Caecal ulcers were significantly higher (*p*= 0.008) in cases with HIV infection, Table 3. Cases without HIV infection suffered more often from complications compared with cases with HIV infection, Table 2.

Our review also identified four asymptomatic cases of GIH in the absence of respiratory tract involvement, as evidenced by normal chest imaging findings [16,32,58,60]. The diagnosis was made based on incidental findings during screening colonoscopy for polyps when ulcerated mucosa [60], ulcerated masses in the ascending colon [58,60] and the entire colon [16,32] were found. Individuals affected were HIV-negative, but immunocompromised, including hepatitis C infection [58], post-liver transplant on immunosuppressive agents [16,32] and psoriatic arthritis on treatment with methotrexate and infliximab [60]. Further evaluation of colonoscopy findings through biopsy of an affected area [16,32,58,60], *Histoplasma* antigen in the urine and antibody to *Histoplasma* antigen [16] further supported the diagnosis of GIH. Treatment with itraconazole resulted in the resolution of the disease [16,32,58,60]. In one patient, a right hemicolectomy was performed as malignancy was suspected [60] before the diagnosis of GIH was made.

Treatment and outcomes were specified in 119 cases. Of the 119,111 (93.3%) had favourable outcomes while eight (6.7%) cases were fatal. Of the 111,35 (31.5%) had HIV infection and of the eight fatal cases, six (75.0%) had HIV infection. The association between positive HIV status and fatal outcomes was statistically significant (*p*= 0.01). Treatment was predominantly with the use of amphotericin B in combination with an azole (n = 70), followed by azole monotherapy (n = 29). Outcomes with the use of combination therapy were favourable in all the cases and 96.6% (28/29) with azole monotherapy (Table 4).

## 4. Discussion

As is known for DH, GIH predominantly occurs in the immunocompromised. However, it can also be found in immunocompetent individuals. GIH mimics several gastrointestinal conditions and is common in many parts of the world, including Africa. It is thus not surprising that despite the increasing cases of DH, GIH is rarely reported. Underreporting is surprisingly pronounced in Africa, a region known to have documented several cases of DH [4,6,125,126,127]. An extensive review on histoplasmosis in Africa that spanned seven decades identified 470 cases of histoplasmosis, of which only one case was reported with GIH [5]. The paucity of data on GIH is first and foremost probably because histoplasmosis is still a neglected fungal disease in most parts of Africa, although with recent epidemiological studies, awareness is improving [125,126,127]. Recognition and reporting of GIH may also be hampered by a lack of adequate diagnostic procedures such as colonoscopy. The use of colonoscopy in the diagnosis of gastrointestinal conditions is still not routine in most African countries. Where such procedures are carried out, there is a dearth of studies on further specialised testing of biopsied samples for *Histoplasma* infection as a likely cause of gastrointestinal changes noted in affected individuals [128,129,130,131]. Prompt targeted evaluation of patients presenting with gastrointestinal disease for histoplasmosis will improve GIH case finding.

The non-specific signs and symptoms of GIH, as highlighted in this review, include but are not limited to abdominal pain [12,18,19,20,20,21,22,25,27,31,33,35,36,37,38,40,41,43,45,47,50,51,54], diarrhoea [12,17,19,21,22,36,37,38,39,40,43,53,68,74,75,103,104,105,106], fever [12,13,17,19,26,27,28,30,31,33,35], weight loss [13,31,77,79,84,98,100,104,111,121], vomiting [12,15,17,27,43,45,50,57,77,99], constipation [69,70,102], haematemesis and haematochezia [18,25,27,29,31,41,44,45,55,59,63,102,105] and associated endoscopic findings, which ranges from intestinal ulcerations [21,24,27,30,33,39,40,41,44,55,69,84,107], strictures [33] and friable masses [12,101] to ulcerated polyps [80]. This plethora of presentations could be misleading and asnon-specific often led to delays in the treatment of patients with GIH.- The initial misdiagnosis was often malignancy [80,83,108,112], inflammatory bowel disease [21,33,40,52,69,89,105,107,116] or acute exacerbation of inflammatory bowel disease for patients already on immunosuppressants [29,30,32,37,53,81] or tuberculosis [85,96,102]. As a result of delay, some patients experienced worsening clinical states [33,69,89,105,107,116] and/or death [20,80,100,116,122]. A high index of suspicion in evaluating GIH in patients is needed as this will reduce the incidence of complications, such as lower gastrointestinal bleeding [17,26,27,29,30,31,33,39,44,53,56,59,63,70,84,94,102,103,105,110,119], intestinal perforation [54,73,80,111], ulceration [70,102,103], intestinal obstruction [15,21,50,57,81,89,95,102,103,104,117], acute pancreatitis [20,65,75,115], acute cholecystitis [44,54,67,68,118] and death [94].

Inflammatory bowel disease (IBD) comprises Crohn’s disease (CD) and ulcerative colitis (UC). It is an autoimmune disorder of the gastrointestinal system, which is managed with immunosuppressive and anti-inflammatory drugs. CD can affect any part of the intestinal wall with a great extent of involvement of the full thickness of transmural mucosa, while UC affects the large colon predominantly with restriction of microscopic findings to the epithelial lining of the gut [132,133]. Presenting complaints of affected individuals are diarrhoea, abdominal pain, bloody stool and vomiting, similar to clinical features seen in individuals with GIH [132,133]. Similarly, the morphological findings during colonoscopy (grossly normal mucosa to oedematous/non-oedematous erythematous mucosa, superficial to deep ulcers with or without lacerations or perforations, strictures and polypoid masses) [84,91], as well as histopathological findings of lymphohistiocytic infiltrates and non-caseating granulomas in patients with GIH also bear semblance to those found in IBD [84,91], and can as well lead to misdiagnosis of GIH cases if deliberate screening for histoplasmosis is not done from the outset [8,105].

The clinicopathological features of GIH also mimic findings seen in intraabdominal malignancies, which can result in a diagnostic mishap if the possibility of *Histoplasma* infection is not explored from the outset [64]. Nine [16,18,33,82,85,103,104,110,114] out of the 123 case reports had presentations mimicking malignancy with treatment targeted at the presumptive diagnosis of malignancy in some of the cases before further evaluation and diagnosis of GIH was done [82,85,110,114]. Five of the patients with malignant mimics had HIV infection with CD4 counts less than 100 [33,82,104,110,114], two were patients on immunosuppressive therapy following lung transplantation (tacrolimus, mycophenolate and steroids) [18,103] and the remaining two were immunocompetent [16,85]. Presenting complaints were based on the site of involvement. Two of the patients had an anal mass and presented with anal pain [16,85] and anal seepage [16]. Lesions at the level of the colon were associated with fever, abdominal pain, [82,103,104,110] and weight loss [33,104,114]. Investigations were done with varied findings, such as an anal mass highly suspicious of neoplasm on pelvic imaging in the absence of other gastrointestinal involvement [16]. Colonoscopy findings were a friable pseudotumoral mass in the sigmoid colon [104], numerous ulcerated sessile polyps throughout the colon [18], oozing umbilicated colonic nodule [33] and ulceronodular growth in the sigmoid colon [82]. Though the gastrointestinal lesions were highly suggestive of malignancy, further evaluation was done for histoplasmosis through histopathology of biopsied specimens from the anus [16,85], colon [18,33,82,103,104] and other resected specimens [104,110], which were suggestive of infection with *Histoplasma capsulatum.* The antibody to *Histoplasma* [18] and urinary *Histoplasma* antigen test [18] were also positive. Treatment was surgery [82,103,110,114] and antifungal agents, including amphotericin B [33,82,104,114] and itraconazole [16,18,33,85,103,104,110,114], with good outcomes as evidenced by resolution of symptoms and regression of gastrointestinal signs. Nevertheless, death was recorded in one patient who also had tuberculosis co-infection [82].

DH can also be complicated with granulomatous hepatitis and/or cholestasis [5]. Thus, when an individual, especially in the setting of an immunocompromised state, presents with fever and jaundice, this disease entity should be considered or ruled out. Of the 123 cases, 16 had granulomatous hepatitis with six having coexisting cholecystitis [22,32,34,47,54,56,61,67,68,72,73,76,83,97,114,118]. Of the 16, 11 were immunocompromised secondary to HIV infection (n = 3) [68,72,114], post-transplantation on immunosuppressive therapy (n = 2) [61,72] and some were on immunosuppressive therapy for various ailments (n = 6), azathioprine [32,76], methotrexate [34,73,118], steroids [32,34,118], infliximab [32,73], fingolimod [54], mycophenolate, adalimumab [76,118], mesalazine [76] and the remainder were immunocompetent [22,47,56,67,97]. Investigations revealed markedly elevated liver enzymes with coexisting gall bladder disorder [22,44,54,67,68,118] and enlarged liver [32,34,68,83]. Diagnosis of granulomatous hepatitis was made via liver biopsy, which showed characteristic features of *H.capsulatum* [22,32,34,54,56,72,73,97]. F confirmatory investigations for histoplasmosis included positive antibody to *Histoplasma* (n = 6) [32,34,67,73,76,98], urinary *Histoplasma* antigen (n = 4) [34,56,67,76], blood culture (n = 3) [47,68,114] and bronchoalveolar lavage staining (n = 1) [114]. Treatment was surgical (cholecystectomy) for individuals with gall bladder involvement [22,68,72], in addition to antifungal therapy with itraconazole and amphotericin B. Marked recovery was noted with a resolution of symptoms and marked reduction in liver enzymes [22,32,34,56,61,67,76,83,114]; death was recorded in one case due to multiorgan failure [72]. *Histoplasma* though an uncommon cause of granulomatous hepatitis is associated with a high mortality rate of 100% [34,56] if not identified and treated.

Though rarely reported, disseminated *Histoplasma* infection can result in acute pancreatitis [20]. Acute pancreatitis is defined as an acute inflammatory process of the pancreas with variable involvement of other regional tissues or remote organ systems [134]. Its clinical manifestation spans from mild abdominal pain to multiple organ failure and sepsis with a high mortality rate [134]. Our review highlights four cases [20,65,77,116] of DH with pancreatic involvement; two were immunocompetent [77,116] while the other two patients were on systemic corticosteroids use [20,65]. The case reported by Asif et al. [20] was a patient with abdominal pain and CT findings of acute pancreatitis in addition to hypertriglyceridaemia. She was managed for acute pancreatitis secondary to hypertriglyceridaemia. Her condition deteriorated with associated complications leading to multiple organ failure and death. Diagnosis of DH with pancreatic involvement was made at post-mortem evaluation. Harris et al. reported an immunocompromised lady on methotrexate and prednisolone for rheumatoid arthritis [65]. She had a history of fever and abdominal pain with increased serum lipase three times the upper limit of normal and an abdominal CT scan of acute pancreatitis. She also had hypertriglyceridaemia. However, given the additional findings of cytopaenias, she had a bone marrow biopsy with histopathological examination done in order to evaluate further for haemophagocytic lymphohistiocytosis during which an incidental finding of *Histoplasma* infection was made and additional serologic testing for the infection was positive. Treatment with amphotericin B and itraconazole resulted in a complete resolution of symptoms [65]. Agrawal et al. [116] and Choudhary et al. [77] reported two cases of CT findings of mass in the head of the pancreas with the common bile duct [116] and duodenal obstruction [77], respectively. Symptoms were jaundice [116], vomiting and progressive weight loss [77]. Diagnosis of GIH was arrived at following endoscopic ultrasound scan guided fine needle aspiration [77] and pancreatectomy [116], with a concomitant histopathological examination, showing small yeasts consistent with *Histoplasma.* A further test for antibody to *Histoplasma* was also positive [77]. Treatment was with itraconazole [116] for one of the patients and voriconazole [77] for the other patient, as she reacted to itraconazole, with improvement of symptoms noted.

With regards to HIV-positive patients, GIH can present in different forms including acute abdomen, co-occurrence with tuberculosis, gastrointestinal bleeding, oesophageal histoplasmosis and intestinal obstruction. Guiot et al. [74] reported the case of a man who presented with acute abdomen seen as severe abdominal pain. He had emergency abdominal surgery, which disclosed a perforated ileum with histopathological findings of the resected segment suggestive of histoplasmosis. A confirmatory test was done with PCR. Additional treatment with antifungals was given with subsequent recovery [74].

The immunocompromised state of individuals with HIV could also result in co-infections where individuals have disseminated histoplasmosis in addition to other opportunistic infections, such as tuberculosis [80,89,100]. Diagnosis of GIH was delayed in one of the patients who was initially evaluated and treated for tuberculosis [100]. The patient’s condition deteriorated with worsening symptoms resulting in death before antifungal treatment could be initiated [100]. The diagnosis was made during post-mortem evaluation [100].

DH with gastrointestinal bleeding due to gastrointestinal involvement is rare [70]. It is seen twice as frequently in HIV/AIDS patients compared with immunocompetent patients [7]. It can cause upper or lower gastrointestinal bleeding depending on the site of involvement [56]. Lower gastrointestinal bleeding may be more common as GIH predominantly affects the ileum and colon as a result of its abundant lymphoid tissue [6]. Lower gastrointestinal bleeding secondary to histoplasmosis is associated with a high mortality rate (20–25%) in those who are immunocompromised [92]. Of the 29 patients with gastrointestinal bleeding, seven were HIV positive [31,41,44,55,71,110,119]. Consideration of GIH in patients with gastrointestinal bleeding is required.

The prevalence of oesophageal histoplasmosis is also higher in individuals with HIV/AIDS, which is said to occur in 3–3.8% of cases [12,13]. Oesophageal histoplasmosis is linked to the early phase of histoplasmosis infection predominantly due to adjoining mediastinal adenitis [6,75] or later from progressive scarring in fibrosing mediastinitis or disseminated disease [6,82]. Endoscopic findings in GIH involving the oesophagus could be erythema or local inflammation [40,42,56,58,105], polyps [13] and ulcerations [20,84,100,104,121]. During the period under review, twelve patients [13,20,40,42,48,56,58,84,100,104,105,121] presented with GIH with oesophageal involvement and five were immunocompromised [13,40,100,104,121] with HIV infection. Treatment with intravenous amphotericin B and itraconazole [13,40,42,48,56,58,84,104,105,121] resulted in a favourable outcome; one patient died due to dual opportunistic infection with tuberculosis [100] and the other died before treatment was commenced [20].

GIH has been identified as a rare cause of intestinal obstruction seen predominantly in people living with HIV/AIDS [50]. This is due to the spectrum of gastrointestinal morphological changes seen in GIH from pseudo-polyps and plaque, ulcerations in the mucosa, stricture formation and thickening of the bowel that affect sites between the duodenum and terminal ileum leading to obstructive symptoms [104]. Past reports of GIH in HIV-infected patients identified that 70% had symptoms of fever and abdominal pain, and 50% had weight loss and diarrhoea [50].

Focusing on cases without HIV infection, the findings from our review suggest the need to also evaluate patients without HIV infection with risk factors for GIH including post-transplant patients on immunosuppressants [16,39,46,49,61,62,63,72,84,101,113], patients on immunosuppressants for other disease conditions [14,15,17,20,25,28,30,69,76,92,103,105] and patients with immunodeficiency syndrome [95] presenting with GI symptoms. This should be done regardless of the immune status of an individual as complications associated with GIH, such as acute abdomen [70], can also develop in immunocompetent patients. Unfavourable outcomes were mainly seen in cases with HIV infection and those receiving amphotericin B deoxycholate monotherapy, and most likely reflect a delayed and more severe clinical disease presentation.

A delayed diagnosis that resulted in the need for surgery was reported as well. Diagnosis of GIH was made following histopathological analysis of a surgically resected specimen [21,33,35,37,60,80,89,94,105,108,109]. Treatment was given with antifungal drugs; thereafter with recovery recorded. However, in the case report by Koh et al. [94] and Seghal et al. [80], the patients died following surgery as a result of delayed diagnosis and treatment for GIH. These cases further buttress the need for a high index of suspicion for GIH in patients presenting with gastrointestinal disease.

## 5. Limitations of the Study

The databases searched for publications included in this review were limited to Pubmed, Google scholar and African Journals Online and so may have missed cases documented in grey literature and other journal articles not available in the searched databases. Moreover, the exclusion of some cases from statistical analysis due to the unavailability of clinical and pathological findings may have influenced the outcome of our analysis.

## 6. Conclusions

GIH occurs with significant frequency in people without HIV infection with similar clinical features and pathological findings as seen in persons living with HIV. The need for an increased index of suspicion in people without HIV infection presenting with GI symptoms and risk factors for GIH is emphasised. In addition, the geographical distribution of GIH cases across the globe suggests a gross underreporting from Africa, despite having a significant at-risk population. Routine screening for histoplasmosis in at-risk patients presenting with gastrointestinal disease will improve GIH case finding.

## Figures and Tables

**Table 1:** Geographical distribution of 212 cases of GIH reported across the globe (2001–2021).

Regions	Country (Number of Cases)	Number of Cases (%)	No. of HIV Positive Patients (%)	No. of HIV Negative Patients (%)	References
North America	USA (n = 67), Mexico (n = 1), Canada (n = 1), Puerto Rico (n = 1)	88 (41.5%)	39 (44.3)	49 (55.7)	[8,12,13,14,15,16,17,18,19,20,21,22,23,24,25,26,27,28,29,30,31,32,33,34,35,36,37,38,39,40,41,42,43,44,45,46,47,48,49,50,51,52,53,54,55,56,57,58,59,60,61,62,63,64,65,66,67,68,69,70,71,72,73,74,75,76]
Asia	India (n = 22), China (n = 4), Taiwan (n = 1), Bangladesh (n = 1), Singapore (n = 2), Malaysia (n = 1), Japan (n = 1)	32 (15.1%)	7 (21.9)	25 (78.1)	[77,78,79,80,81,82,83,84,85,86,87,88,89,90,91,92,93,94,95,96,97,98]
South America	French Guiana (n = 65), Ecuador (n = 6), Brazil (n = 4), Peru (n = 2), Argentina (n = 1), Trinidad and Tobago (n = 1)	79 (37.3%)	72 (91.1)	7 (8.9)	[99,100,101,102,103,104,105,106,107,108,109,110,111]
Europe	Netherlands (n = 2), United Kingdom (n = 2), Austria (n = 1), Switzerland (n = 1), Portugal (n = 1), Spain (n = 1)	8 (15.1%)	3 (37.5)	5 (62.5)	[112,113,114,115,116,117,118,119]
Central America	Honduras (n = 1), Guatemala (n = 1)	2 (0.9%)	2 (100)	-	[120,121]
Australia	Australia (n = 2)	2 (0.9%)	-	2 (100)	[122,123]
Africa	Ivory Coast (n = 1)	1 (0.5%)	1 (100)	-	[124]
Total		212	124	88	

**Table 2:** Comparison between GIH cases with and without HIV infection.

Characteristics	HIV Infection (%)	No HIV Infection (%)	*p*-Value
n = 123	41 (33.3)	82 (66.7)	
**Symptoms/findings**			
Abdominal pain	27 (65.9)	34 (41.5)	0.01
Fever	20 (48.8)	36 (43.9)	0.7
Weight loss	19 (46.3)	32 (39.0)	0.4
Diarrhoea	15 (36.6)	30 (36.6)	1
Passage of blood in stool	9 (22.0)	16 (18.2)	0.8
Generalised body weakness	6 (14.6)	12 (19.5)	0.8
Splenomegaly	3 (7.3)	10 (12.2)	0.5
Anorexia	7 (17.1)	9 (11.0)	0.4
Vomiting	9 (22.0)	9 (11.0)	0.1
Night sweats	6 (14.6)	12 (14.6)	1
Hepatomegaly	4 (9.8)	9 (11.0)	1
Nausea	10 (24.4)	9 (11.0)	0.07
Jaundice	2 (4.9)	4 (4.9)	1
Dysphagia	1 (2.4)	2 (2.4)	1
Haematemesis	1 (2.4)	2 (2.4)	1
Odynophagia	4 (9.8)	2 (2.4)	0.09
Constipation	1 (2.4)	2 (2.4)	1
Anal lesion	2 (4.9)	2 (2.4)	0.6
Bloating	0	1 (1.2)	1
**Site of infection**			
Colon	19 (46.3)	35 (42.7)	0.7
Small bowel	16 (39.0)	20 (24.4)	0.1
Gastric cavity	5 (12.2)	6 (7.3)	0.5
Oesophagus	5 (12.2)	7 (8.5)	0.5
Liver	5 (12.2)	14 (17.1)	0.3
Gall bladder	2 (4.9)	6 (7.3)	0.6
Pancreas	0	4 (4.9)	0.3
Anus	0	2 (2.4)	0.6
**Complications**			
Gastrointestinal bleeding	11 (26.8)	18 (22)	0.7
Hepatitis	5 (12.2)	14 (17.1)	0.6
Intestinal Obstruction	7 (17.1)	8 (9.8)	0.3
Cholecystitis	2 (4.9)	5 (6.1)	1
Acute pancreatitis	0	4 (4.9)	0.3
Acute abdomen	2 (4.9)	2 (1.6)	0.6
Bowel perforation	7 (17.1)	5 (6.1)	0.1
**Mode of diagnosis**			
Histopathology	37 (90.2)	72 (87.8)	0.8
Urinary *H. capsulatum* antigen	7 (17.1)	14 (17.1)	1
Culture	6 (14.6)	11 (13.4)	1
Serum *H. capsulatum* antibody	2 (4.9)	2 (2.4)	0.6
Serum *H. capsulatum* antigen	1 (2.4)	7 (8.5)	0.3
*H. capsulatum* real-time PCR	2 (4.9)	3 (3.7)	1

**Table 3:** Endoscopic findings.

Characteristics	HIV Infection (%)	No HIV Infection (%)	*p*-Value
n = 84	28 (33)	56 (66.7)	
**Site of involvement**	**Ulcers**		
Caecum	9 (32.1)	4 (7.1)	0.008
Ascending colon	2 (7.1)	7 (12.5)	0.7
Transverse colon	3 (10.7)	4 (7.1)	0.7
Descending colon	1 (3.6)	0	0.3
Sigmoid colon	2 (7.1)	2 (3.6)	0.6
Rectum	1 (3.6)	6 (10.7)	0.4
Entire colon	0	7 (12.5)	0.09
Undefined	1 (3.6)	6 (10.7)	0.4
Terminal ileum	8 (28.6)	9 (16.1)	0.2
Duodenum	2 (7.1)	2 (3.6)	0.6
Oesophagus	3 (10.7)	2 (3.6)	0.3
Gastric cavity	3 (10.7)	2 (3.6)	0.3
**Erythematous mucosa /Inflammation**			
Caecum	0	2 (3.6)	0.6
Ascending colon	1 (3.6)	1 (1.8)	1
Transverse colon	0	3 (5.4)	0.5
Descending colon	0	1 (1.8)	1
Sigmoid colon	0	1 (1.8)	1
Rectum	0	2 (3.6)	0.6
Entire colon	0	2 (3.6)	0.6
Undefined	0	0	-
Terminal ileum	0	2 (3.6)	0.6
Duodenum	1 (3.6)	0	0.3
Oesophagus	1 (3.6)	4 (7.1)	0.7
Gastric cavity	2 (7.1)	4 (7.1)	1
Nodular/Polypoid			
Caecum	2 (7.1)	5 (8.9)	1
Ascending colon	2 (7.1)	3 (5.4)	1
Transverse colon	2 (7.1)	1 (1.8)	0.3
Descending colon	1 (3.6)	0	0.3
Sigmoid colon	3 (10.7)	1 (1.8)	0.1
Rectum	0	2 (3.6)	0.6
Entire colon	0	0	-
Undefined	0	4 (7.1)	0.3
Terminal ileum	0	2 (3.6)	0.6
Duodenum	2 (7.1)	0	0.1
Oesophagus	1 (3.6)	0	0.3
Gastric cavity	0	0	-

n = total number of patients that had an endoscopic procedure.

**Table 4:** Summary of treatment and outcomes.

Treatment	Number of Cases	Favourable Outcome	Death
AmB + Azoles	70	70	-
AmB alone	11	6	5
LAmB	1	1	-
LAmB + Azoles	1	1	-
Azole monotherapy	29	28	1
Azole + Terbinafine	1	1	-
Surgery	2	2	-
AmB + Surgery	1	-	1
Azole + Surgery	1	1	-
AmB + Antiviral medications	2	1	1
Total	119	111	8

AmB; amphotericin B, LAmB; liposomal amphotericin B.

## Data Availability

Data sharing is not applicable to this article.
